# Improvement in prescriptions while maintaining overall health outcomes: a prospective observational study conducted in Japanese facilities for older people

**DOI:** 10.1186/s12877-022-02872-4

**Published:** 2022-04-13

**Authors:** Takumi Ashizawa, Sari Mishina, Ataru Igarashi, Tsukasa Kobayashi, Yoshimasa Takase, Shunya Ikeda

**Affiliations:** 1 Department of Health Economics and Outcomes Research, Graduate School of Pharmaceutical Sciences, the University of Tokyo, 7-3-1 Hongo, Bunkyo-ku, Tokyo, 113-0033 Japan; 2 Unit of Public Health and Preventive Medicine, Yokohama City University School of Medicine, 3-9 Fukuura, Kanazawa-Ku, Yokohama, Kanagawa 236-0004 Japan; 3 Life Co., Ltd., Tennozu Central Tower F18, 2-2-24 Higashi-Shinagawa, Shinagawa-Ku, Tokyo, 140-0002 Japan; 4 Takase Clinic, 1-5-1-5F, Yaguchi, Ohta-Ku, Tokyo, 146-0093 Japan; 5 Department of Public Health, School of Medicine, International University of Health and Welfare, 4-3 Kozunomori, Narita-shi, Chiba, 286-8686 Japan

**Keywords:** Polypharmacy, Potentially inappropriate medication, Quality of life, Activities of daily living, Cognition, Medication costs

## Abstract

**Background:**

Improvements in the use of polypharmacy or potentially inappropriate medication (PIM) may reduce medication costs in Japan. We aimed to evaluate the impact of improvement in prescription on both overall health outcomes and medication costs in Japanese facilities for older people.

**Methods:**

Residents in Japanese facilities for older people between March 2019 and March 2020 were included in this study. The following five indices were used to evaluate overall health outcomes: EuroQoL-5D-5L, Barthel Index, Mini Mental State Examination, Dementia Behaviour Disturbance Scale, and Vitality Index. The team, which consisted of one physician and several pharmacists, suggested improved prescriptions for the attending physicians of the participants. The impact of improvement in prescriptions on the health outcomes score, medication costs, and the number of medications were evaluated through two comparison groups: those whose number of medications decreased (decrement group, DG) and those whose medications did not (not decrement group, NDG); those prescribed PIMs in March 2019 and those not prescribed PIMs in March 2020 (improvement group, IG) and those prescribed PIMs both in March 2019 and March 2020 (not improvement group, NIG). In both comparison groups, propensity score matching was performed to balance demographics, and all health outcome scores, medication costs, and the number of medications in March 2020 were assessed using a t-test. Statistical significance was set at a *p*-value of < 0.05.

**Results:**

Eight-hundred-and-ninety-one participants (75.5% women, 86.2 ± 7.9 years old) were enrolled. After matching, in the comparison between the DG (*N* = 232, 77.2%, 85.7 ± 8.5) and NDG (*N* = 232, 78.5%, 86.0 ± 3.1), changes in the health outcomes score were nonsignificant. However, the medication costs and the number of medications significantly decreased. After matching, in the comparison between IG (*N* = 141, 75.2%, 86.7 ± 8.1) and NIG (*N* = 273, 74.2%, 86.2 ± 8.3), changes in health outcome scores and medication costs were not significant. However, the number of medications significantly decreased.

**Conclusions:**

Improvements in prescriptions did not adversely affect the overall health outcomes. However, it impacted medication costs and the number of medications. Improvement in prescriptions could decrease medication costs while maintaining overall health outcomes.

## Background

Polypharmacy and potentially inappropriate medication (PIM), which is induced by polypharmacy, are crucial issues for older people worldwide [1]. Polypharmacy is defined as the use of multiple drugs simultaneously and is often defined as the use of five or more drugs in combination per day [2]. PIM is a medication that has a higher risk than its anticipated benefits [3, 4]. In 2016, there were 14 different criteria for PIMs, such as the Beers criteria and the STOPP/START criteria, and 729 different medications/classes reported in all criteria. Most criteria for PIMs include the usage of benzodiazepines, NSAIDs, antihistamines and antipsychotics for older people [5]. Several studies reported that the use of polypharmacy or PIMs increased the risk of adverse events and hospitalization [6–8]. Older people often use polypharmacy or PIMs because they often suffer from a combination of diseases and have multiple physiological dysfunctions. Systematic reviews regarding the use of PIMs have reported that approximately 11.5–62.5% of older people used PIMs [9, 10].

The use of polypharmacy and PIMs are serious issues in Japan, one of the most aging societies in the world. The proportion of people aged 65 years or older is 28.7%in March 2020 and this is estimated to reach 30.0% by 2025 [11–13]. With rapid ageing, national medical care expenditure in Japan is increasing, and as of 2018, it was Japanese yen (JPY) 43.4 trillion, which is estimated to reach JYP 66.7–68.5 trillion in 2040 [14, 15]. Increasing the number of prescriptions or the number of days per prescription was one of the causes for the increase in national medical care expenditures [16]. Based on the data reported by Suzuki et al., 33.2% of people over 65 years in Japan are prescribed five or more medicines, and 22.9% are using PIMs [17]. Therefore, it is expected that improving the use of polypharmacy or PIMs will lead to a reduction in national medical care expenditures.

However, the value of appropriate use of medication should not only be considered in terms of costs. The fundamental concept of the cost-effectiveness of health care interventions is that both costs and health outcomes should be simultaneously analysed [18, 19].

A few studies on polypharmacy or PIMs have been conducted in Japan, and almost all of them take only single components, health outcomes or costs, into account. A study using the data of hospitalized older patients showed that people prescribed six or more medications were at a higher risk of adverse events than those who were prescribed five or fewer medications [20]. A study has been conducted in Japan to evaluate the impact of improving the appropriate use of polypharmacy or PIMs on quality of life (QoL) and activities of daily living (ADL) [21]. Previous studies also reported that medication costs decreased with improvements in prescriptions, and the amount was JPY 65.6–170.4 per day [22, 23].

The purpose of this study was to simultaneously evaluate the impacts of improvement in prescriptions on both overall health outcomes and medication costs and to ensure that this improvement would lead to decreased medication costs while maintaining overall health outcomes.

## Methods

### Study design

This was a prospective observational study. Participants enrolled in this study were admitted to nursing homes or residential facilities with health and caregiving services for older people under Life Company Limited, Tokyo, Japan, between March 2019 and March 2020 (47 facilities, *N* = 3461). A team of one physician and several pharmacists cooperating with Life Company Limited proposed optimized prescriptions for each resident to attending physicians. This proposal project had already started in June 2018 in one facility, and it expanded every 3 months to other facilities in sequence. This proposal was supervised by the author YT who is one of the co-authors of this paper and participated in developing the guidelines for the appropriate use of medications for older people [24].

The prescriptions that residents received were collected, and surveys were conducted in March 2019 and March 2020. Using their prescriptions, daily costs of medications and the number of medications were estimated. The price of each medication was derived from the “National drug tariff in Japan 2020” [25]. Overall health outcomes were assessed in March 2019 and March 2020 by staff working at the facilities using the following five measurements: EuroQoL-5D-5L-proxy (EQ-5D-5L), Barthel Index (BI), Mini-Mental State Examination (MMSE), Dementia Behaviour Disturbance Scale (DBD), and Vitality Index (VI). Demographics of participants, such as age and sex, were derived from the database managed by Life Company Limited.

All residents in the facilities whose informed consent was obtained participated in this study, and participants whose demographic data (age, sex, five health outcome indices and prescription) was obtained in March 2019 and whose data were still available 1 year later were included in the analysis.

### Questionnaires

#### EuroQoL-5D-5L-proxy (EQ-5D-5L)

The EQ-5D-5L was used to evaluate health-related QoL. This consisted of the following five dimensions: “Mobility,” “Self-Care,” “Usual Activities,” “Pain/Discomfort,” and “Anxiety/Depression.” Answers for each dimension were merged and converted to the QoL score, in which 0.0 indicated death and 1.0 indicated perfect health. Negative QoL scores (below 0) were assigned to extremely bad health status [26, 27]. The Japanese tariff was used for the conversion from original answers to QoL scores [28].

#### Barthel index (BI)

BI was used to assess the ADL. Using BI, ADL were measured on a scale of 0 to 100, with higher scores indicating greater independence from physical assistance [29]. A score of 60 appeared to be a pivotal score where patients transitioned from dependency to assisted independence, and for those with a Barthel score below 40, it was found that none had independent mobility skills, and fewer than 50% were independent in very basic skills, such as feeding, grooming, and sphincter control [30].

#### Mini-mental state examination (MMSE)

The MMSE is one of the most popular questionnaires used to measure cognitive function [31]. It was also used to assess the severity of dementia. The maximum score that could be achieved in the MMSE was 30. By using the MMSE, patients with MMSE scores of 21–23, 11–20, and 0–10 were classified as suffering from mild, moderate, and severe Alzheimer’s disease, respectively [32, 33].

#### Dementia behaviour disturbance scale (DBD)

Behavioural disturbance is a common and distinctive feature of dementia. DBD was used to assess the severity of behavioural disturbance, and the severity of behavioural disturbance was measured on a scale of 0 to 112, with increases in DBD scores indicating more severe behavioural disturbance [34]. There were two types of DBD: one consisted of 28 questions, and the other consisted of 13 questions [35]. The former was used in this study.

#### Vitality index (VI)

VI was developed to measure vitality related to ADL in older patients with dementia. Using VI, vitality was measured on a scale of 0 to 10, with higher scores indicating more vitality related to ADL [36].

### Comparisons

The following three comparisons were conducted in this study to compare health outcome scores (EQ-5D-5L, BI, MMSE, DBD, and VI), daily costs of medications, and the number of medications.

#### Comparison in terms of the facilities where suggestions for improving prescriptions were conducted

Participants were classified into two groups according to the timing of when proposals for improving prescriptions were started at the facilities they resided in. Those who were in facilities where the proposals were implemented by the end of March 2020 (31 facilities) were categorized into the implemented facilities group (IFG), and the rest were categorized into the not implemented facilities group (NIFG). It should be noted that this classification was based on facilities, not based on whether participants underwent a decrease in medications.

#### Comparison in terms of the number of decreased prescribed medications

Participants whose number of prescribed medications in March 2020 decreased compared to that in March 2019 were categorized into the decreased group (DG). Residents who had increased or no change in the number of prescribed medications were categorized into the not decreased group (NDG).

#### Comparison in terms of the improvement in the use of PIMs

According to the “Guidelines for medical treatment and its safety in older people 2015,” PIMs were referred to as cautiously administered medications being prescribed to older people with a high risk of adverse events [37]. Participants who were prescribed PIMs in March 2019 and not prescribed it in March 2020 were categorized into the improvement group (IG), and those who were prescribed PIMs both in March 2019 and March 2020 were categorized into the not improvement group (NIG).

### Data analyses

To neglect the effects of extraordinarily high-priced anticancer drugs on the daily costs of medication, participants whose daily cost of medication was over ten thousand JPY were omitted as outliers. Fixed ratio propensity score matching was conducted in March 2019 to balance demographics such as age, sex, health outcomes score, daily costs of medications, and the number of medications [38, 39]. After matching, the health outcomes score, daily costs of medications, and the number of medications in March 2020 were assessed using a t-test [40]. Statistical significance was set at *p*-value < 0.05, and all analyses were performed using Python version 3.7.7.

## Results

**Fig. 1:**
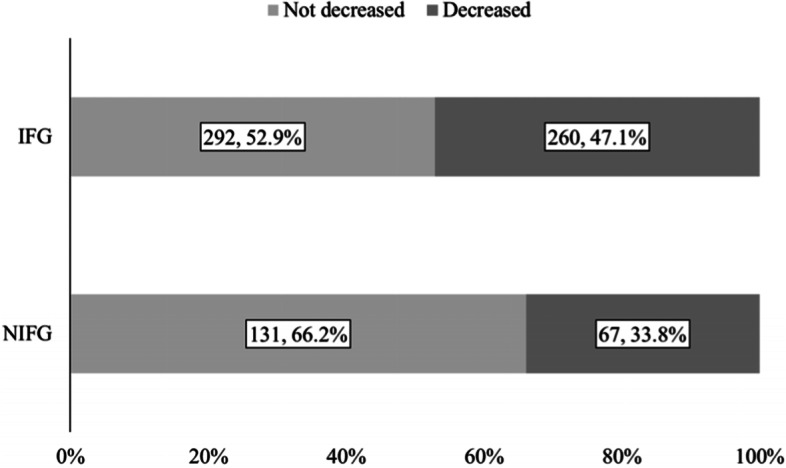
Distribution of the participants with a decrease in the number of medications. IFG (implemented facilities group), participants who were in facilities where proposals for improving inappropriate prescriptions were implemented by the end of March 2020 were classified into this group. NIFG (not implemented facilities group), participants who were not in facilities where proposals for improving inappropriate prescriptions were implemented by the end of March 2020 were classified into this group. *The number of people and their proportion are written in squares. **Summation of the number of people in the figure is not consistent with 889, which was the target population for analysis because there were participants with missing data in March 2020

**Fig. 2:**
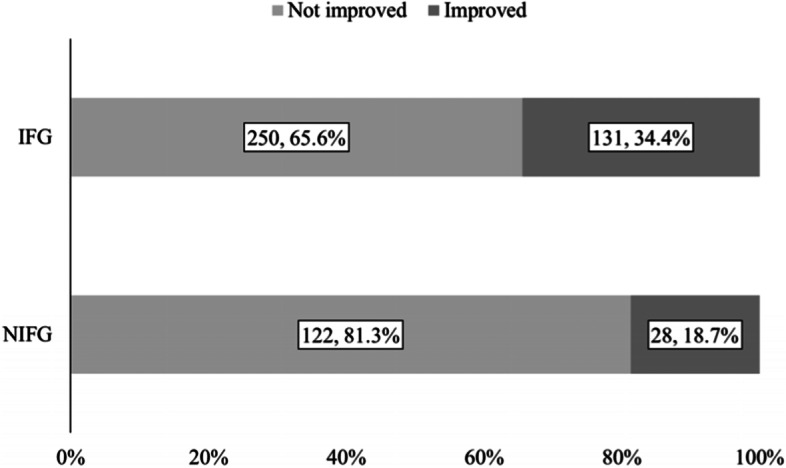
Distribution of participants with an improvement in the use of PIMs. IFG (implemented facilities group), participants who were in facilities where proposals for improving inappropriate prescriptions were implemented by the end of March 2020 were classified into this group. NIFG (not implemented facilities group), participants who were not in facilities where proposals for improving inappropriate prescriptions were implemented by the end of March 2020 were classified into this group. *The number of people and their proportion are written in squares. **Summation of the number of people in the figure is not consistent with 889, which was the target population for analysis. This was because a few participants did not use PIMs or there were participants with missing data in March 2020

Both questionnaires and prescriptions were collected from 1260 participants in March 2019. Out of the 1260 participants, 891 participants (75.5% women, 86.2 ± 7.9 years old) who were still available 1 year later were included in the analysis. Other demographics, such as types of nursing care level, medications received, health outcomes score, daily costs of medications, and the number of medications, are shown in Table 1. The proportion of participants whose medications were reduced increased by 13.3 pts. (IFG: 47.1%, NIFG: 33.8%), and that of participants who stopped using PIMs increased by 15.7 pts. (IFG: 34.4%, NIFG: 18.7%) (Figs. 1 and 2). Decreased medications in the prescriptions in the DG group included magnesium oxide, rebamipide, and amlodipine besylate, while those in the IG group were magnesium oxide, furosemide, and spironolactone (Tables 2 and 3).

**Table 1:** Demographics of the participants whose data were analysed in March 2019

Number of people	891
Sex (Women, %)	75.5%
Age (mean ± SD, years)	86.15 ± 7.89
Nursing care level^a^ (nursing care level > 2, %)	37.5%
EQ-5D-5L scores (mean ± SD)	0.63 ± 0.25
BI scores (mean ± SD)	61.9 ± 32.8
MMSE scores (mean ± SD)	17.0 ± 8.8
DBD scores (mean ± SD)	16.2 ± 13.8
VI scores (mean ± SD)	7.5 ± 2.6
Daily medication costs (mean ± SD, JPY/day)	507.1 ± 629.6
Number of medications (mean ± SD)	7.1 ± 3.7
Concomitant drugs (Usage, %)	
Antidiabetic	11.7%
Antihypertensive	16.0%
Antihyperlipidaemic	38.8%
Antiulcer	47.6%
Vasodilator	44.7%
Antacid	44.1%

*SD* standard deviation; *EQ-5D-5L* EuroQoL-5D-5L-proxy, *BI* Barthel Index, *MMSE* Mini-Mental State Examination, *DBD* Dementia Behaviour Disturbance Scale, *VI* Vitality Index

^a^ The degree of care needed in Japan is divided into seven categories: two “support needed” levels plus five “nursing care” levels. The type of care services provided under a long-term care insurance scheme in Japan is determined based on these categories

**Table 2:** Medications with decreased consumption found in the number of people in the DG

	Number of people taking medications		Difference	Percentage
Generic name	A. 2019	B. 2020	C. A-B	C/A
Magnesium Oxide ^a^	170	118	52	30.6%
Levamipide	38	6	32	84.2%
Amlodipine Besil	118	88	30	25.4%
Heparinoid	49	20	29	59.2%
Loxoprofen ^a^	36	16	20	55.6%
Ketoprofen ^a^	30	11	19	63.3%
Dimethylisopropyl azulene	29	11	18	62.1%
Sennoside	72	54	18	25.0%
White Vaseline	31	13	18	58.1%
Ambroxol	22	5	17	77.3%
Acetaminophen	41	26	15	36.6%
Donepezil	53	39	14	26.4%
Famotidine ^a^	23	9	14	60.9%
Betamethasone butyrate propionate	18	4	14	77.8%
Mosapride	19	5	14	73.7%

^a^ Generic name listed in the “Guidelines for medical treatment and its safety in the older people 2015”

DG (decrement group), the participants whose number of medications decreased during the study period (between March 2019 and March 2020)

**Table 3:** Medications with decreased consumption found in the number of people in the IG

	Number of people taking medications		Difference	Percentage
Generic name	A. 2019	B. 2020	C. A-B	C/A
Magnesium Oxide^a^	118	43	75	63.6%
Furosemide^a^	57	40	17	29.8%
Spironolactone^a^	41	25	16	39.0%
Sennoside	41	28	13	31.7%
White Vaseline	17	5	12	70.6%
Butyric acid bacteria	24	13	11	45.8%
Amlodipine	57	47	10	17.5%
Dimethylisopropyl azulene	15	5	10	66.7%
Famotidine^a^	13	3	10	76.9%
Azithromycin	9	0	9	100.0%
Ambroxol	11	2	9	81.8%
Ketoprofen^a^	13	4	9	69.2%
Loxoprofen^a^	16	7	9	56.3%
Liver-Inhibiting Powder plus Tangerine Peel, Pinellia Tuber	9	0	9	100.0%
Oseltamivir	8	0	8	100.0%

^a^ Generic name listed in “Guidelines for medical treatment and its safety in older people 2015”

IG (improvement group), the participants who were prescribed PIMs in March 2019 and those who were not prescribed PIMs in March 2020

The study period was between March 2019 and March 2020

### Comparison in terms of the facilities where suggestions for improving prescriptions were conducted (IFG vs. NIFG)

Before matching, the number of participants in the IFG was 604 and that in the NIFG was 273. After 1:2 matching, the number of participants in each group was 459 and 234, respectively. The demographics of each group are shown in Table 4. There were no significant changes in any health outcome score, daily costs of medications or the number of medications (Table 5).

**Table 4:** Comparison between the demographics of the NIFG and that of the IFG before and after matching

	NIFG	IFG	Standardized difference
Before matching			
Number of people	273	604	
Sex *women, %*	72.9	76.7	0.084
Age *years, mean ± SD*	87.1 ± 7.2	85.7 ± 8.2	0.18
EQ-5D-5L scores *mean ± SD*	0.63 ± 0.26	0.63 ± 0.25	0.012
BI scores *mean ± SD*	59.3 ± 33.7	63.2 ± 32.2	0.12
MMSE scores *mean ± SD*	16.0 ± 9.5	17.4 ± 8.4	0.15
DBD scores *mean ± SD*	17.6 ± 14.1	15.6 ± 13.6	0.14
VI scores *mean ± SD*	7.4 ± 2.7	7.50 ± 2.6	0.038
Medication costs *JPY/day, mean ± SD*	429.8 ± 504.8	542.1 ± 706.1	0.18
Number of medications *mean ± SD*	7.0 ± 3.5	7.10 ± 3.7	0.041
After matching			
Number of people	234	459	
Sex *women, %*	72.2	75.6	0.075
Age *years, mean ± SD*	86.7 ± 7.3	87.0 ± 7.0	0.037
EQ-5D-5L scores *mean ± SD*	0.64 ± 0.27	0.64 ± 0.25	0.0010
BI scores *mean ± SD*	61.2 ± 34.0	61.3 ± 32.4	0.0040
MMSE scores *mean ± SD*	16.7 ± 9.5	16.5 ± 8.3	0.012
DBD scores *mean ± SD*	16.2 ± 13.3	16.3 ± 14.0	0.0083
VI scores *mean ± SD*	7.4 ± 2.7	7.5 ± 2.6	0.020
Medication costs *JPY/day, mean ± SD*	451.1 ± 534.2	457.0 ± 515.2	0.011
Number of medications *mean ± SD*	7.0 ± 3.5	6.8 ± 3.7	0.045

NIFG (not implemented facilities group), participants who were not in facilities where proposals for improving inappropriate prescriptions were implemented by the end of March 2020 were classified into this group

IFG (implemented facilities group), participants who were in facilities where proposals for improving inappropriate prescriptions were implemented by the end of March 2020 were classified into this group

**Table 5:** Results of the comparison between the NIFG and IFG in March 2020

	NIFG	IFG	t-value	*p*-value
Number of people	232	455		
EQ-5D-5L scores *mean ± SD*	0.66 ± 0.50	0.64 ± 0.46	0.48	0.63
BI scores *mean ± SD*	56.6 ± 34.7	54.8 ± 33.9	0.62	0.54
MMSE scores *mean ± SD*	14.7 ± 10.5	14.4 ± 9.4	0.25	0.80
DBD scores *mean ± SD*	16.7 ± 12.9	17.0 ± 13.4	−0.21	0.83
VI scores *mean ± SD*	7.2 ± 2.9	7.1 ± 2.9	0.41	0.68
Medication costs *JPY/day, mean ± SD*	466.9 ± 393.3	474.7 ± 566.9	− 0.16	0.87
Number of medications *mean ± SD*	6.9 ± 3.0	6.5 ± 3.3	1.38	0.17

*SD* standard deviation, *EQ-5D-5L* EuroQoL-5D-5L-proxy, *BI* Barthel Index, *MMSE* Mini-Mental State Examination, *DBD* Dementia Behaviour Disturbance Scale, *VI* Vitality Index

NIFG (not implemented facilities group), participants who were not in facilities where proposals for improving inappropriate prescriptions were implemented by the end of March 2020 were classified into this group

IFG (implemented facilities group), participants who were in facilities where proposals for improving inappropriate prescriptions were implemented by the end of March 2020 were classified into this group

### Comparison in terms of the decrease in the number of medications (DG vs. NDG)

Before matching, the number of participants in the DG was 321 and that in the NDG was 416. After 1:1 matching, the numbers of participants in each group were 232 and 232, respectively. The demographics of each group are shown in Table 6. There were no statistically meaningful changes in health outcomes. However, the changes in the number of medications and the daily costs were statistically significant. The mean values of the number of medications for DG and NDG were 5.0 and 9.1, respectively. The daily costs for each group were 351.8 and 728.4, respectively (Table 7).

**Table 6:** Comparison between demographics of the NDG and that of the DG before and after matching

	NDG	DG	Standardized difference
Before matching			
Number of people	416	321	
Sex *women, %*	74.52	78.82	0.097
Age *years, mean ± SD*	86.1 ± 7.9	86.28 ± 7.93	0.017
EQ-5D-5L scores *mean ± SD*	0.64 ± 0.25	0.61 ± 0.24	0.12
BI scores *mean ± SD*	61.9 ± 32.8	60.5 ± 31.4	0.043
MMSE scores *mean ± SD*	16.5 ± 8.9	17.3 ± 8.7	0.089
DBD scores *mean ± SD*	16.6 ± 13.7	16.4 ± 14.5	0.018
VI scores *mean ± SD*	7.4 ± 2.6	7.5 ± 2.5	0.024
Medication costs *JPY/day, mean ± SD*	434.1 ± 638.6	615.8 ± 712.8	0.27
Number of medications *mean ± SD*	5.7 ± 3.1	8.8 ± 3.4	0.96
After matching			
Number of people	232	232	
Sex *women, %*	78.5	77.2	0.032
Age *years, mean ± SD*	86.0 ± 8.1	85.7 ± 8.5	0.034
EQ-5D-5L scores *mean ± SD*	0.60 ± 0.26	0.60 ± 0.25	0.010
BI scores *mean ± SD*	58.5 ± 33.5	57.8 ± 32.9	0.021
MMSE scores *mean ± SD*	16.7 ± 9.6	16.7 ± 9.1	0.0055
DBD scores *mean ± SD*	16.2 ± 12.9	16.9 ± 14.7	0.046
VI scores *mean ± SD*	7.3 ± 2.8	7.3 ± 2.6	0.014
Medication costs *JPY/day, mean ± SD*	548.3 ± 700.3	593.1 ± 811.2	0.059
Number of medications *mean ± SD*	7.5 ± 2.8	7.6 ± 2.8	0.026

*SD* standard deviation, *EQ-5D-5L* EuroQoL-5D-5L-proxy, *BI* Barthel Index, *MMSE* Mini-Mental State Examination, *DBD* Dementia Behaviour Disturbance Scale, *VI* Vitality Index

NDG (not decrease group), participants who had increased or no change in the number of prescribed medicines were classified into this group

DG (decrease group), participants in whom there was a decrease in the number of prescribed medicines in March 2020 compared to that of March 2019 were classified into this group

**Table 7:** Results for the comparison between the NDG and DG in March 2020

	NDG	DG	t-value	*p*-value
Number of people	232	232		
EQ-5D-5L scores *mean ± SD*	0.64 ± 0.60	0.64 ± 0.44	0.036	0.97
BI scores *mean ± SD*	51.2 ± 34.5	53.8 ± 32.4	−0.75	0.46
MMSE scores *mean ± SD*	13.9 ± 9.9	15.2 ± 10.0	−1.01	0.32
DBD scores *mean ± SD*	16.8 ± 12.4	17.2 ± 12.8	−0.28	0.78
VI scores *mean ± SD*	6.9 ± 3.0	7.1 ± 2.9	−0.53	0.59
Medication costs *JPY/day, mean ± SD*	728.4 ± 619.7	351.8 ± 520.2	7.02	*p* < 0.001*
Number of medications *mean ± SD*	9.1 ± 3.3	5.0 ± 2.5	15.04	*p* < 0.001*

* Statistically significant

NDG (no decrease group), participants who had increased or no change in the number of prescribed medicines were classified into this group

DG (decrease group), participants in whom there was a decrease in the number of prescribed medicines in March 2020 compared to that of March 2019 were classified into this group

### Comparison in terms of the improvement in the use of PIMs (IG vs. NIG)

Out of the 891 participants, 521 (58.4%) used PIMs in March 2019. Before matching, the number of patients in the IG was 153, and that of the NIG was 368. After 1:2 matching, the number of participants in each group was 141 and 275, respectively. The demographics of each group are shown in Table 8. There were no statistically significant changes in any health outcomes or daily costs of medications. However, the number of medications significantly decreased, and the mean values for IG and NIG were 6.0 and 7.9, respectively (Table 9).

**Table 8:** Comparison between demographics of the NIG and that of the IG before and after matching

	NIG	IG	Standardized difference
Before matching			
Number of people	368	153	
Sex *women, %*	75.00	74.51	0.011
Age *years, mean ± SD*	86.4 ± 7.9	86.6 ± 8.0	0.025
EQ-5D-5L scores *mean ± SD*	0.62 ± 0.26	0.59 ± 0.24	0.13
BI scores *mean ± SD*	60.2 ± 32.2	58.0 ± 33.1	0.067
MMSE scores *mean ± SD*	17.0 ± 8.9	16.0 ± 8.6	0.12
DBD scores *mean ± SD*	16.5 ± 14.3	17.9 ± 14.5	0.10
VI scores *mean ± SD*	7.4 ± 2.6	7.3 ± 2.6	0.055
Medication costs *JPY/day, mean ± SD*	499.0 ± 598.0	601.1 ± 656.2	0.16
Number of medications *mean ± SD*	7.6 ± 3.4	8.5 ± 3.8	0.27
After matching			
Number of people	275	141	
Sex *women, %*	74.2	75.2	0.023
Age *years, mean ± SD*	86.2 ± 8.3	86.7 ± 8.1	0.063
EQ-5D-5L scores *mean ± SD*	0.59 ± 0.26	0.59 ± 0.24	0.026
BI scores *mean ± SD*	57.7 ± 33.4	57.9 ± 33.2	0.070
MMSE scores *mean ± SD*	16.2 ± 9.2	16.2 ± 8.5	0.0023
DBD scores *mean ± SD*	18.0 ± 14.8	17.9 ± 14.3	0.071
VI scores *mean ± SD*	7.3 ± 2.7	7.3 ± 2.6	0.027
Medication costs *JPY/day, mean ± SD*	523.1 ± 460.3	560.0 ± 629.6	0.067
Number of medications *mean ± SD*	8.1 ± 3.4	8.3 ± 3.8	0.059

*SD* standard deviation, *EQ-5D-5L* EuroQoL-5D-5L-proxy, *BI* Barthel Index, *MMSE* Mini-Mental State Examination, *DBD* Dementia Behaviour Disturbance Scale, *VI* Vitality Index

NIG (not improvement group), those who were prescribed both in March 2019 and March 2020 were classified into this group

IG (improvement group), participants who were prescribed and administered medications cautiously in March 2019 and not prescribed in March 2020 were classified into this group

**Table 9:** Results of the comparison between the NIG and IG in March 2020

	NIG	IG	t-value	*p*-value
Number of people	275	141		
EQ-5D-5L scores *mean ± SD*	0.59 ± 0.30	0.69 ± 0.74	−1.72	0.087
BI scores *mean ± SD*	54.2 ± 33.6	50.0 ± 34.7	1.05	0.29
MMSE scores *mean ± SD*	14.0 ± 9.7	13.6 ± 9.4	0.30	0.77
DBD scores *mean ± SD*	17.3 ± 13.2	175. ± 12.5	−0.14	0.89
VI scores *mean ± SD*	7.2 ± 2.9	6.7 ± 3.1	1.24	0.22
Medication costs *JPY/day, mean ± SD*	546.2 ± 536.8	495.0 ± 483.5	0.94	0.35
Number of medications *mean ± SD*	7.9 ± 3.4	6.0 ± 3.2	5.27	*p* < 0.001*

* Statistically significant. NIG (not improvement group), those who were prescribed both in March 2019 and March 2020 were classified into this group

IG (improvement group), participants who were prescribed and administered medications cautiously in March 2019 and not prescribed in March 2020 were classified into this group

## Discussion

Few studies have assessed both medication costs and overall health outcomes in the same study. The results of our study showed that improving prescriptions would not adversely affect the overall health outcomes, and this contributed to a decrease in the number of medications and medication costs.

The reason why a decrease in medications was observed was partially due to the use of PIMs. Some medications that should be cautiously administered to older people (e.g., magnesium oxide, loxoprofen and ketoprofen) became less likely to be prescribed. However, the usage of other medications, such as levamipide and moisturizer (heparinoid), which were not classified into PIMs, was also decreased (Tables 2, 3). This could be interpreted as the avoidance of over-prescriptions.

### Comparison with previous research

In previous studies, QoL and ADL were not impaired by reducing medications or improving PIMs [41, 42]. Moreover, cognitive function was not found to be impaired [43]. The results of our study were consistent with these results. In all comparisons in this study, none of the indices of health outcomes showed statistically meaningful changes (Tables 5, 7, and 9).

When comparing the IFG and NIFG, a decrease in medication costs was not observed (Tables 4 and 5). This result is inconsistent with previous research, which reported that medication costs decreased by JPY 65.6 per day. This could have occurred because more participants used PIMs in the previous research than the participants in this study. Ohshima et al. observed that 76.9% of the participants used PIMs [22]. This proportion was higher than that found in this study (58.4%).

In the comparison between the DG and NDG, the participants in whom the use of polypharmacy improved, medication costs significantly decreased (Table 7). In a previous study similar to this comparison (comparing whether the number of medications decreased or not), it was reported that medication costs decreased by JPY 170.4 per day, and this was consistent with the result of this study, which showed that medication costs decreased by JPY 241.3 per day (Tables 6 and 7) [23].

We could consider a hypothetical situation in which a proposal for improving prescriptions is implemented for the 710,000 older people in Japan who reside in facilities for older people as of 2018 [44]. In the IFG group, there was a 13.3% increase in the number of participants who had a decrease in the number of medications compared to the NIFG group, and daily medication costs were found to decrease by JPY 241.3 per day (Fig. 1, Tables 6 and 7). Given these results, for 710,000 older people, the number of medications may be reduced for 94,430 older people, and the annual medication costs may be reduced by JPY 8.3 billion.

### Limitations

There are five main limitations of this study. The first limitation is representativeness. This study was conducted in private facilities for older people, where many older people who needed care lived due to illnesses or functional disorders. The participants in this study may be in a worse state of health than the general older people individuals in Japan. In addition, the difference between private and public facilities should be considered, as it affects representativeness. The admission criteria for public facilities tend to be stricter than those of private facilities, which means that residents in public facilities tend to have worse health statuses than those in private facilities. The out-of-pocket expenditure of public facilities is less than that of private facilities, which implies the possibility that the income of residents in private facilities is different from that of residents in public facilities. Moreover, in this study, the baseline data of 1260 out of 3461 (36.4%) participants were completely collected in March 2019. Nonresponse biases might have also occurred, even though propensity matching balanced the baseline between comparison groups.

The second limitation is that some residents who participated in this study in March 2019 moved out of the residential facilities by March 2020 due to hospitalizations or a change in their place of residence. The impact of improvements in prescriptions on overall health outcomes and medication costs might not have been evaluated precisely because some of the residents who were participants moved out.

The third limitation is that the improvement period was different after the development of the proposals because the proposals were launched sequentially at each facility every 3 months from June 2018. In particular, the study result of the comparison between the IFG and NIFG might be underestimated for the participants in the facilities where proposals had been conducted before the beginning of this study.

The fourth limitation is the lack of long-term efficacy data, laboratory data and subjective data. The long-term impact, such as over 1 year, was not assessed sufficiently, and it was difficult to evaluate the minor changes in laboratory data, such as HbA1C, and subjective components of a health state, such as pain.

The fifth limitation is that the demographics of participants, especially past medical history, were not sufficiently obtained, and it was difficult to determine the use of PIMs. Essentially, PIMs should be determined by considering not only the type of medication but also the history of the patient. Therefore, it is likely that the proportion of people using PIMs was overestimated and the impacts of improvement in the use of PIMs were underestimated because it was only classified by the type of medications.

The strength of our study is that it evaluates the impact of improving prescriptions on overall health outcomes and costs simultaneously. Improvement in prescriptions must not be promoted only to reduce medication costs, without considering its impact on overall health outcomes. There were some limitations, one of which was that this study was conducted in nursing home care facilities. Further studies that are conducted under other situations, such as public facilities or in-home-based care, are warranted. Despite some limitations, we believe that the results of this study have important implications for promoting improvement in prescriptions and could facilitate appropriate prescriptions while considering cost-effectiveness.

## Conclusions

Modification of the use of polypharmacy and/or PIMs would decrease medication costs and the number of medications prescribed while maintaining one’s overall health outcomes. Further studies, which could facilitate appropriate prescriptions while considering cost-effectiveness, are warranted.

## Data Availability

The datasets generated and analysed during the current study are not publicly available due to informed consent documents but are available from the corresponding author on reasonable request.

## Abbreviations

PIM: Potentially Inappropriate Medications
QoL: Quality of Life
ADL: Activities of Daily Living
JPY: Japanese Yen
EQ-5D-5L: Euro-QoL-5D-5L
BI: Barthel Index
MMSE: Mini-Mental State Examination
DBD: Dementia Behaviour Disturbance Scale
VI: Vitality Index
IFG: Implemented facilities group
NIFG: Not implemented facilities group
DG: Decreased Group
NDG: Not decrease group
IG: Improvement Group
NIG: Not Improvement Group
